# Poly(2-oxazoline)s as Stimuli-Responsive Materials for Biomedical Applications: Recent Developments of Polish Scientists

**DOI:** 10.3390/polym14194176

**Published:** 2022-10-05

**Authors:** Aleksandra Lusina, Tomasz Nazim, Michał Cegłowski

**Affiliations:** Faculty of Chemistry, Adam Mickiewicz University in Poznań, Uniwersytetu Poznańskiego 8, 61-614 Poznań, Poland

**Keywords:** 2-oxazolines, poly(2-oxazoline)s, stimuli-responsive materials, drug delivery systems

## Abstract

Poly(2-oxazoline)s are the synthetic polymers that are the products of the cationic ring-opening polymerization (CROP) of 2-oxazoline monomers. Due to their beneficial properties, from which biocompatibility, stealth behavior, high functionalization possibilities, low dispersity, stability, nonionic character, and solubility in water and organic solvents should be noted, they have found many applications and gained enormous interest from scientists. Additionally, with high versatility attainable through copolymerization or through post-polymerization modifications, this class of polymeric systems has been widely used as a polymeric platform for novel biomedical applications. The chemistry of polymers significant expanded into biomedical applications, in which polymeric networks can be successfully used in pharmaceutical development for tissue engineering, gene therapies, and also drug delivery systems. On the other hand, there is also a need to create ‘smart’ polymer biomaterials, responsive to the specified factor, that will be sensitive to various environmental stimuli. The commonly used stimuli-responsive biomedical materials are based mostly on temperature-, light-, magnetic-, electric-, and pH-responsive systems. Thus, creating selective and responsive materials that allow personalized treatment is in the interest of the scientific world. This review article focuses on recent discoveries by Polish scientists working in the field of stimuli-responsive poly(2-oxazoline)s, and their work is compared and contrasted with results reported by other world-renowned specialists.

## 1. Introduction

**Figure 1:**
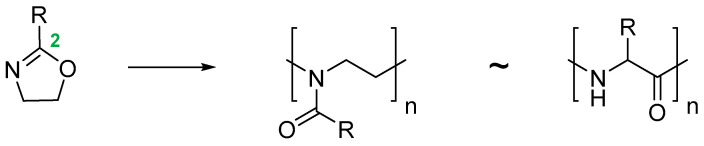
Polymerization of 2-oxazolines and their structural similarity with poly(amino acid)s.

For the last few decades, polymer chemistry has significantly expanded into biomedical applications. Polymer networks can be easily found within biomedical fields, in which they can be used as polymeric biomaterials applied in tissue engineering, polymer therapeutics, and implant technology. Thus, creating selective and responsive materials that allow personalized treatment is in the interest of the scientific world. As a result, there is a need for creating materials with supramolecular platforms that will provide a wide range of well-defined pharmaceutics systems with high functionalization possibilities [1]. Due to the fact that polymer networks are in possession of this required feature and allow the obtention of highly defined materials to build complex pharmaceutics systems, they are commonly used [2]. As described, the biocompatible polymers have gained researchers’ interest and become useful in drug delivery systems in a number of complex applications. These polymers might be directly attached to pharmaceuticals and thus can be a part of the drug substance. The subsequent conjugation to lipids will allow it to become a part of the liposomal delivery system, whereas, by linking with cationic molecules, they can become a part of the nucleic acid complexing polymer. As there are some examples of well-defined biostable and biodegradable polymers widely used in medicine such as poly(ethylene glycol) (PEG) or *N*-(2-hydroxypropyl)methacrylamide (HPMA), another class of high-quality polymers is polyoxazolines [3]. Most researchers are focusing on the polymerization of 2-substituted monomers [4,5], because of the fact that the 4- and 5-position of oxazoline’s monomers are more difficult to polymerize due to steric crowding [6]. The poly(2-oxazoline)s are thus the products of cationic ring-opening polymerization (CROP) of 2-oxazoline monomers. The polymerization of 2-oxazolines, resulting in poly(2-alkyl/aryl-2-oxazoline)s (PAOx), has been reported by four independent research groups [7,8,9,10] as a result of the publishing 10 years earlier of the first ‘living’ and controlled polymerization reaction by Szwarc [11,12]. The obtained PAOx is an organic class of poly(amide)s that can be regarded as pseudo-peptides due to their structural similarities to naturally occurring peptides [13] (Figure 1).

The main difference is that the peptide bonds present within the peptide structure link together repeating units that come from amino acids, whereas the pseudo-peptide bonds from PAOx link a substituent with the polymer main chain. It is a fact that the interaction between an amide group and a non-polar side chain (substituent) makes PAOx able to show side-chain-dependent properties [14].

The obtained biocompatibility, stealth behavior, nonionic character, stability, high functionalization possibilities, low dispersity, and solubility in water and organic solvents have caused poly(2-oxazoline)s to find many applications [1,3,15,16]. In response to the reported properties with the connection of high versatility attainable by copolymerization or post-polymerization modification, this class of polymeric systems has been widely used as a polymeric platform for novel biomedical applications [17,18].

## 2. Synthesis of 2-Oxazoline Monomers

**Figure 2:**
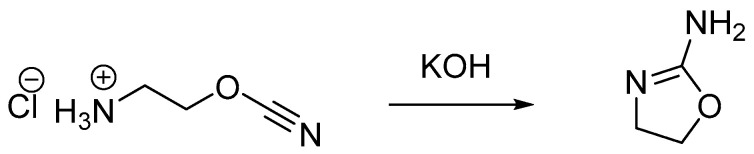
Synthesis of the first reported 2-oxazoline monomer derivative: 2-amino-2-oxazoline.

As has already been mentioned, the 4- and 5- substituted oxazoline monomer derivatives are not well polymerizable. On the other hand, the 2-substituted 4,5-dihydrooxazoles, called 2-oxazolines, are commonly used cyclic imino ethers for a CROP reaction [19]. The first synthesized 2-oxazoline monomer, 2-amino-2-oxazoline, was prepared in 1889 by the isomerization of the free base of 2-cyanoethyl-1-ammonium chloride [20]. The overview of the synthesis of the first reported 2-oxazoline monomer derivative is presented in Figure 2.

**Figure 3:**
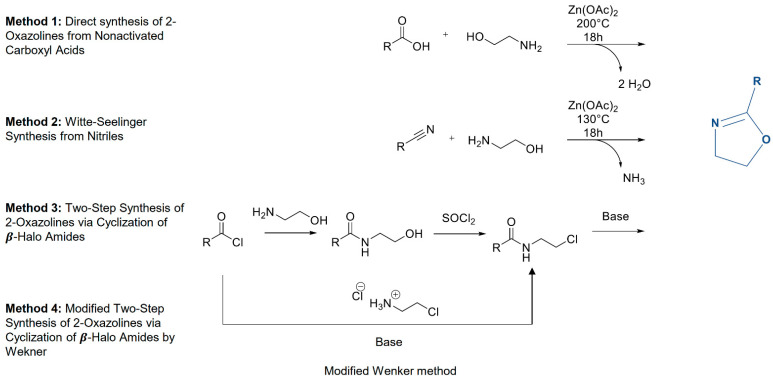
The representation of possible routes for 2-oxazoline monomer synthesis. Adapted from [19].

Due to the presence of a nucleophilic amine group within the obtained 2-oxazoline derivative, this monomer is not able to be used for the subsequent CROP reaction. The presence of any nucleophilic function within the monomer structure leads to the spontaneous termination of polymerization at an early stage or even causes interference with the initiation process. Thus, the presence of, for instance, alcohols, thiols, or unprotected amines must be avoided in the 2-oxazoline derivatives that are planned to be polymerized. The additional factor that should be discussed is the influence of the substitution in 4- or 5- positions to the CROP. In general, the presence of 4- or 5- substitutions slows down polymerization because of the steric barriers [21]. However, the 4- and 5-substituted oxazolines are polymerizable, which is commonly used in asymmetric catalysis, wherein monomers with 5-substitution (or even 4-substitution) can be used to increase the stability of the ligands [22], whereas, from the first 2-oxazoline monomer synthesis in 1889, a variety of strategies were reported, allowing 2-oxazoline derivatives to be obtained. This paragraph will be focused only on the methodologies that allow the obtention of the monomer derivatives capable of further CROP reaction. The possible monomer synthesis pathways were combined in one graph and are presented in Figure 3.

## 3. The Cationic Ring-Opening Polymerization (Crop) of 2-Oxazoline Monomers

**Figure 4:**
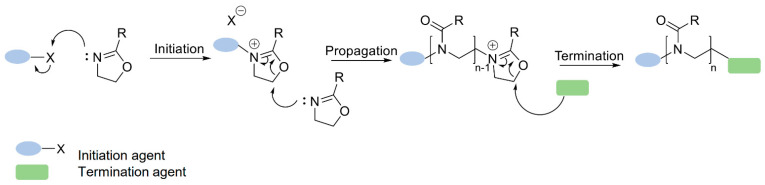
General mechanism of the living cationic ring-opening polymerization (CROP) of 2-oxazoline monomers. Adapted from [12].

As mentioned previously, the poly(2-oxazoline)s are the products of the living cationic ring-opening polymerization (CROP) reaction of 2-oxazoline monomers [19]. The CROP follows a typical chain-growth polymerization mechanism including initiation, propagation, and termination, which is presented step by step in Figure 4. The figure presents the main steps of the polymerization reaction: initiation by electrophilic medium addition, propagation caused by nucleophilic substitution, and final termination with the nucleophile chemical compound [12].

In accordance with literature reports, the CROP reaction of 2-oxazoline monomers can be processed in a living or quasi-living manner under appropriate conditions [12]. From the thermodynamic point of view, the CROP reaction is unfavorable due to the value of the entropy of polymerization, whereas the driving force of the process is the enthalpic gain caused by the isomerization of the cyclic imino ether into a more stable tertiary amide. The result is a spontaneous polymerization of 2-oxazoline monomers after initiation, often performed at elevated temperature conditions [23,24]. Additionally, to obtain living polymerization, the purity of every component plays an important role. It is crucial to use the extreme purity and dryness of solvents, as well as initiators and monomers, to reach optimal polymerization conditions. Due to the fact that the presence of nucleophilic species in the polymerization mixture causes the irreversible termination step, these molecules are not allowed. Furthermore, during the polymerization reaction, the absence of strong bases is required, as these species induce the deprotonation of the cation, which subsequently leads to the promotion of chain transfer reactions. By maintaining the described rules, it is possible to reach the living mechanisms of the polymerization reaction. Thus, the post-polymerization modification of poly(2-oxazoline) derivatives creates an important area for further research [25,26]. In the following subsections, each of the polymerization steps shown in Figure 4 (initiation, propagation, and termination) will be described.

### 3.1. Initiation

**Figure 5:**
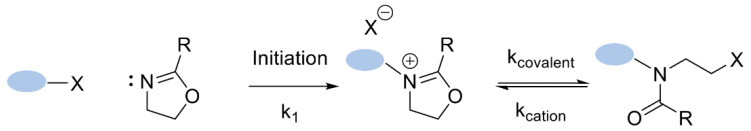
The initiation of the living cationic ring-opening polymerization (CROP) of 2-oxazoline monomers. Equilibrium between the cationic and covalent molecules depends on the counter ion. Adapted from [12].

The initiation step is an exothermic reaction running with an initiation constant rate (k_1_). It occurs as a nucleophilic attack of the cyclic imino ethers (2-oxazoline monomers) on the electrophilic initiator agent, which directly results in the formation of an oxazoline cation (Figure 5) [12].

As the data show, there is a variety of initiators that can be successfully used to determine the CROP of 2-oxazoline monomers. The initiator can be both organic and inorganic Lewis acids [7,10], chloroformates [27], silyl halides [24], various alkylating agents (alkyl halide, tosylates, and triflates), and also supercritical CO_2_ [28]. Currently, the most useful initiators seem to be alkylating agents, from which the methyl triflate, methyl tosylate, methyl iodide, and benzyl bromide should be mentioned. Thanks to the high chemical and physical stability, methyl tosylate is therefore the most preferred CROP initiator [29]. As mentioned, the initiation is usually performed at elevated temperatures. However, the CROP can also ben performed at room temperature by using extremely reactive electrophiles, such as the mentioned triflates [29]. Compared to this data, even under elevated temperatures, some of the reported initiators exhibit slow initiation such as ((2-oxo-1,3-dioxolan-4-yl)methyl 4-methyl benzenesulfonate [30] or 3-butynyl tosylate [31]. As a result of low efficiency or slow initiation, the desired first-order kinetics of polymerization will not be achieved, which will subsequently lead to broader molar mass distribution [12].

During the initiation process, the counter ion present in the mixture influences the equilibrium between the cationic molecule and the covalent molecule of the 2-oxazoline monomer defined as k_covalent_ and k_cation_ (Figure 3). These are the equilibrium-rate constants for the interconversion of the cationic into the covalent molecule and vice versa, depending on the utilized initiator, monomer, and solvent [32]. The obtained covalent molecule is a result of the ring-opening isomerization of the 2-oxazoline monomer and plays an important role in the subsequent propagation step [33].

It should be noticed that, at the initiation step, the functionalities can be introduced within the poly(2-oxazoline)-derivatives structure. This strategy requires the consideration of the functional groups present in the initiator molecule, which needs to be compatible with the CROP of 2-oxazoline monomers and should also provide basic advantages, such as fast initiation, to avoid reaching high molar-mass distributions. The techniques of introducing end-group functionalities are well-known and have been widely reported in the literature [34,35,36]. Furthermore, at this step of CROP, the shape of the final polymer can be established. In literature, there are several reports that describe obtaining star-shaped [37,38], graft, or even comb copolymers [39] by using 2-oxazoline monomers in CROP. As a result, initiation can be summarized as a step of huge importance for obtaining the desired polymers by their shape or content of functional groups.

### 3.2. Propagation

**Figure 6:**
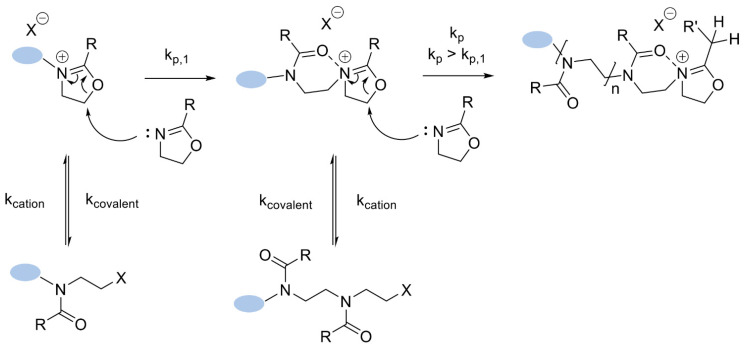
The propagation mechanism of the living cationic ring-opening polymerization (CROP) of 2-oxazoline monomers. Adapted from [12].

The propagation reaction is more complex, as it consists of a two-step mechanism. The general mechanisms of each step of this reaction are presented in Figure 4. As it shows, the first step is based on the addition of the first monomer to the product obtained during the initiation (the rate is determined by the constant k_p,1_). Because of the slow rate, this step can be a determining step, if the full initiation process is ensured [33]. As also shown in Figure 6, during this step, an equilibrium between the cationic and the covalent species is established, which is determined by the counter ion, solvent, and monomer. The equilibrium present in this step can be compared with the equilibrium discussed within the initiation process part. After achieving the total initiation efficiency and completing the first step of propagation, the second step is taking place. This step is determined by the increased propagation rate constant—k_p_—established by the neighboring dipole-ion polarization effect. This polarization effect can be explained as a result of the stabilization of the obtained transition state and the subsequent shifts of the equilibrium to the more reactive cationic species, which is presented in Figure 6. The shifts of the equilibrium are the results of the influence of various parameters, such as temperature, concentration, or even solvent [33,40]. At this step, again, the equilibrium between the cationic and covalent species plays an important role, as the k_p_ shows a linear relationship and is dependent on the percentage of the cationic species of 2-oxaline monomers [33,41]. The conclusion made at this step can be summarized as the cationic species playing an enormous role and should be treated as the responsible individual for the propagation process.

The most important parameters that have a strong impact on the k_p_ value within the CROP of 2-oxazoline monomers are the monomer, temperature, and solvent. It has been reported that, for the polymerization reactions that do not consist of cationic 2-oxazoline monomers propagating species, the obtained equilibrium between cationic and covalent propagating individuals is shifted with the increasing polarity of the used solvent. It is well-known that polar solvents are helpful and cause the stabilization of the cationic species by solvation. As a result, it leads to an acceleration of the CROP reaction. One of the most valuable examples was reported in 1998 by a Polish scientist, Professor Dworak, who investigated the influence of the solvents on the efficiency of the CROP reaction. In this study, the 2-methyl-2-oxazoline monomer (MeOx) was initiated by benzyl bromide, which resulted in the obtention of 62 +/- 4% of cationic propagating species in the highly polar solvent (nitrobenzene) and, in the comparison, less than 3% of cationic propagating species in a less polar solvent (tetrachloromethane) [32]. The studies were based on the article from 1976, reported by Professor Saegusa, which described the first reported MeOx polymerization by using the mentioned benzyl bromide as an initiator agent [42]. The proposed studies, which were the prelude to Professor Dworak’s experiments, used the CH_3_CN as a solvent medium. In fact, as the data showed, most of the current research performs the CROP of 2-oxazoline monomers using acetonitrile at 140 °C [12]. Due to the fact that the boiling point of the acetonitrile is 82 °C, the proposed temperature conditions (140 °C) caused the creation of overpressure, which can be easily achieved using a closed reaction vessel, such as a microwave oven [43]. However, the use of a microwave oven is not required, because the CROP can be performed by using superheated conventionally pressure reactors. Both of the reported methodologies allow the obtention of the same polymers; thus, there are no specific microwave effects, and the results show the same kinetics [44].

As mentioned previously, the monomer structure is also an important factor that shapes the k_p_ of the 2-oxazoline monomers’ CROP reaction. Generally, the k_p_ value can be modulated by the side-chain modification of the used 2-oxazoline derivative. As the 2-oxazoline monomers form a wide group of compounds, they are prone to various modifications, and thus, the modification’s products can include a wide range of functional groups. Finally, these functionalities may lead to the broader dispersity of the obtained poly(2-oxazoline)s derivatives or, on the other hand, of no polymerization due to the potential interference of the built-in groups to the CROP mechanism [45]. Some of the functional groups that have an impact on the CROP mechanism and thus, the chain transfer, are presented in Table 1.

The structure of the monomer affects the partial positive charge present in the 5-position of the cationic 2-oxazoline propagating species and the nucleophile character of the imine functionality of the monomer. In fact, these two factors are contrary to each other. It has been shown that donating an electron to the 2-oxazoline monomer causes an increase in the nucleophilicity of the imine functionality, which makes it more reactive. At the same time, the partial cationic charge of the 5-positions from the 2–oxazoline monomer propagation species decreases, which makes it less reactive. Additionally, it should be noted that the steric effects also play an important role and shape the k_p_ value, which makes it much more difficult to predict. However, studies are still being performed [46].

### 3.3. Termination

Corresponding to the previous CROP stages, the termination process is also dependent on the termination rate constant: k_t_, which is determined by the equilibrium between the cationic and covalent species. Due to the fact that the growing chain ends of the polymer are relatively stable cations, the termination process is often performed using harsh conditions (for example, by using high-temperature conditions combined with carboxylate anions as terminating agents) [47]. However, the termination conditions of 2-oxazoline monomer polymerization depend on the electrophilicity of the cation and the reaction partners. Thus, the termination can be performed not only in harsh conditions, but sometimes there is a possibility of performing it under mild conditions. For example, the reported hydroxide species or cyclic amines terminating at room temperature can be noted [48]. Other reported examples are thiols, which require minimal excess and no elevated temperature [3], and azides, which can be used at both room [49] and elevated temperatures [50]. 

**Figure 7:**
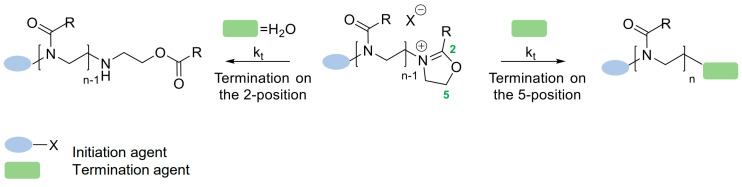
The termination mechanisms of the living cationic ring-opening polymerization (CROP) of 2-oxazoline monomers. Adapted from [12].

The termination of the CROP of (2-oxazoline)-based polymers occurs by the nucleophilic attack in the 2- and 5- positions, which results in ester terminated polymer chains or the introduction of the end group, respectively [12]. The general process of the termination step is presented in Figure 7.

**Figure 8:**
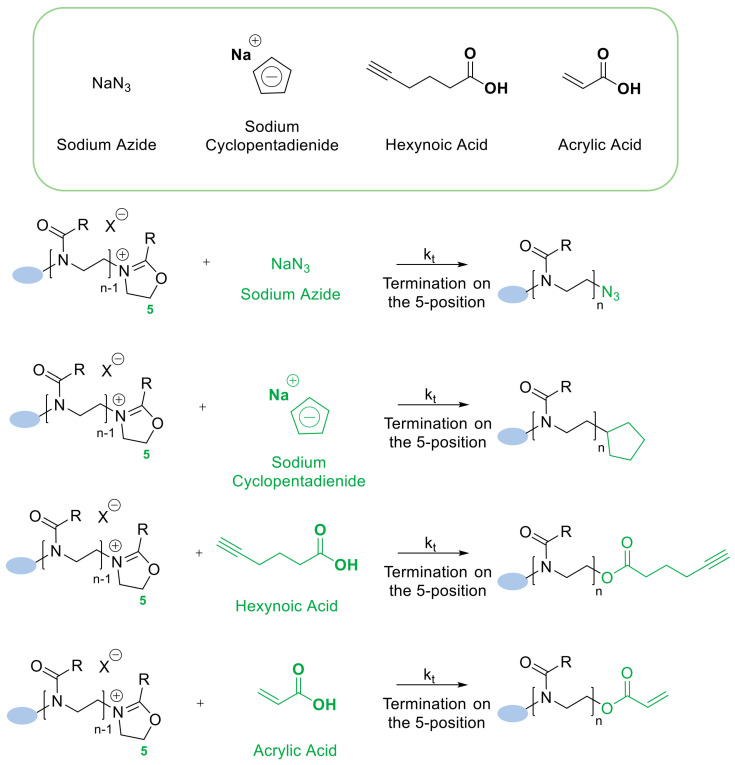
An overview of the chemical structures of end-functionalities that may be introduced by the termination step of CROP and the general termination mechanism with using these terminating agents.

The termination step can be used for introducing the desired functionalities into the polymer structure. As mentioned previously, the initiation step allows the modification of the structure, thus both of these processes provide a possibility of introducing a variety of end groups. There are three main classes of terminating agents, used during the termination step. The first class is a hydroxyl end-group functionality that has been reported to proceed through a OH-nucleophilic attack to the 5-position. The most described example of hydroxyl end-group functionality is the methanolic KOH solution [48]. The second class of commonly used terminating agents is a wide range of nitrogen-based compounds. While the use of ammonia had been thought to generate the primary amine-end group [51], recent investigations led by a Polish scientist prove that a termination with ammonia leads to hydroxyethyloamino end groups [52]. The important fact is that the use of primary and secondary amines results in, respectively, secondary and tertiary amine end-groups [53], and the use of tertiary amines yields a quaternary ammonium end-group [54]. Additionally, one of the most popular end-group modifications of poly(2-oxazoline)-based materials is the nucleophilic substitution made by azides. As reported, the azides have been widely used and applied in poly(2-oxazoline)s functionalization [51]. The third, and last class of terminating agents are carboxylate derivatives, widely reported to introduce end-groups such as styrene derivatives, acrylates [52], or even methacrylates [53]. It is also well-known that besides the mentioned classes of terminating agents, the thiols represent potential nucleophile agents that can be used in the termination step during CROP reaction [51]. Generally, many end-functionalities are suitable for post-polymerization modification using click chemistry and can be easily introduced at the termination step. However, the number of them is limited, and the most popular agents are presented in Figure 8 [26].

## 4. The Biological Application Context of Poly(2-oxazoline)s

According to the thesis that poly(2-oxazoline) derivatives seem to be ideal candidates for pharmaceutical applications, there is a need to focus on their biocompatibility. However, biocompatibility is a wide complex purpose, and it is based on the interaction of the material with a variety of biological entities (for example, proteins or membranes). The possible interactions formed inside the organism may be based on hydrophobic interactions, hydrogen bonding, electrostatic, or any other. It means that there is a necessity for creating tailor-made pharmaceutics [54]. While the main application of the polymer-based drug seems to be the release of the encapsulated drug over time by diffusion, there are also some complex multifunctional polymers with covalently attached drug moieties constructed. The use of polymers in combination with drug molecules possesses several significant advantages that make these materials more useful than the pure drug molecule. These mentioned advantages are improved pharmacogenetics, reduced antigenic activity, increased solubility of insoluble drugs or drugs of low aqueous solubility, the possibility of combination with other functional components such as contrast agents, and protection against deactivation and degradation during transport [55]. Whereas the majority of well-investigated polymers for biomedical applications have focused on poly(ethylene oxide) (PEO) [17], there are also lots of reports showing the biocompatibility of poly(2-methyl-2-oxazoline) (PMeOx) [56] and poly(2-ethyl-2-oxazoline) (PEtOx) [57]. The reported beneficial features of PMeOx—biocompatibility, stealth behavior, and biodistribution—made this polymer useful in biomedical applications. Despite the more hydrophilic character than PEO [58], which might cause complications in nonpolar organic solvents, PMeOx shows great potential for use in pharmaceutical applications. Nevertheless, there is still a necessity to perform in-depth studies of the poly(2-oxazoline)s to estimate the possible degradation pathways. Due to the reported higher cytotoxicity of the copolymers of 2-ethyl-2-oxazoline and ethylene imine than PEtOx [59], there is a need for detailed studies of the potential formation of poly(ethylene imine) residues by the enzymatic degradation of the amine bonds of poly(2-oxazoline)s [60].

One of the most important parameters that should be considered in order for drug delivery to have a favorable performance is an estimation of the behavior of the used compound in the physiological environment. It is not surprising that modern drug delivery systems (DDS) must be stable in physiological media and also should show the release behavior only in specific conditions without any side effects. The necessity of using selective and sensitive materials that are able to produce personalized and high-performance treatments caused the joining of forces between medicine and polymer science. This interdisciplinary research caused poly(2-oxazoline)-based DDS to gain popularity over the past years, due to the possibility of the stimuli-responsive feature. The proposed materials have utilized the common fact that physiological stimuli (temperature, pH, redox, etc.) are generally used for release behavior, which is strictly connected with the values of these physiological parameters that differ between inflammatory and normal tissue. It is worth mentioning that most of the examples of poly(2-oxazoline)-based DDS have been reported over the few past years and that the number of biomedical application of these compounds are still relatively small in comparison with other types of DDS [61].

## 5. Poly(2-oxazoline)s as Stimuli-Responsive Polymers

Polymers that undergo reversible or irreversible changes as a result of interactions with non-destructive, small environmental changes including heat, light, magnetic or electrical field, pH, etc., are called stimuli-responsive polymers. 

**Figure 9:**
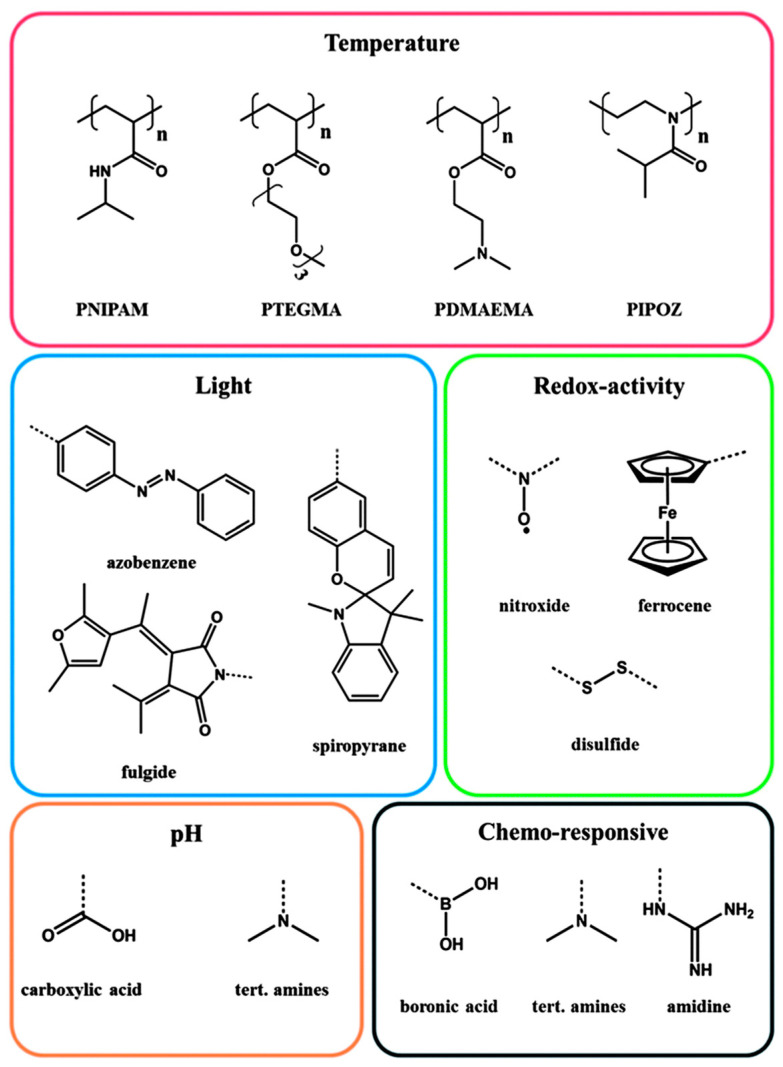
Examples of stimuli-responsive functional groups. Reprinted from [67]. Copyright (2022), with permission from Elsevier [or applicable society copyright owner].

They belong to a group of materials characterized by their adaptability to the surrounding environment because of their response to external factors. This responsive behavior elicited by an external stimulus can be manifested in a variety of ways. The changes that can occur in polymer chains are most often the result of the breakage or formation of weak intermolecular interactions (hydrogen, π–π, dispersive, electrostatic). These changes can be expressed primarily in the conformational changes of individual polymer chains and differences in interactions with other polymers or solvent molecules (resulting in a change in solubility). Similar interactivity has been originally observed in biological systems, wherein macromolecular compounds such as proteins and nucleic acids play an active, responsive role. Designing stimuli-responsive polymers for biomedical applications is therefore one of the most important challenges of biomimicry. Examples of functional groups, sensitive to specific external stimuli, are shown in Figure 9 [62,63,64,65,66].

Designing responsive polymer-based systems includes incorporating monomeric units that are sensitive to environmental changes. Macroscopic structures such as hydrogels, micelles, or polymer brushes are often made up of complex block copolymer chains, the responsive behavior of which is related to the presence of components that are capable of changing their properties under external stimuli. Changes in individual monomeric units, which include, for instance, protonation (or deprotonation) in varying environmental pH, bond breakage under the influence of temperature or changes in conformation under the influence of light are factors that affect the entire polymeric structure [68,69,70].

There are also many examples referring to the synthesis of multifunctional copolymers, containing more than one for the stimuli-responsive functional group. This concept of structural design increases the variety of potential applications and allows the creation of more sophisticated systems that, in theory, are more selective in terms of stimulus response. The controlled drug delivery in the immediate vicinity of cancer cells can be mentioned as an illustrative example, since the tumor cells’ milieu has a different physiological pH and temperature than healthy tissues [67]. The numerous applications of stimuli-responsive polymers include, among others, fields such as sensing, controlled drug delivery, actuation, tissue engineering, catalysis, and dispersion-stabilizing agents. In these sectors, stimuli-responsive polymers are becoming more and more significant [68,71,72,73,74]. 

Poly(2-oxazoline)s are fully suitable for biological applications. From the structural point of view, this polymer family can be considered pseudo-peptides. Compared to other biocompatible polymers, such as PEG, poly(2-oxazoline)s are characterized by lower toxicity, faster decomposition time, and flexibility regarding the synthesis of the desired structure (their properties can be easily tuned with the implementation of various functional groups) [3,75]. 

In terms of cytotoxicity and hemocompatibility, poly(2-ethyl-2-oxazoline) was investigated as an alternative for PEG [76]. Both polymers turned out to be highly tolerated in short-term experiments, even at high doses. Some cytotoxic effects, as the study revealed, occurred only after prolonged incubation at concentrations greater than therapeutic dosages. The numerous advantages of poly(2-oxazoline)s over PEG include primarily: high stability at room temperature, low viscosity, relatively high drug loading, and high removal rate (tested on mice, wherein no accumulation in tissues was observed, whereas PEG may accumulate in vivo) [3]. 

The most frequently reported applications of poly(2-oxazoline)s are primarily due to their properties described above, including the controlled delivery of molecules (drugs, proteins DNA, RNA) in living organisms. Among these mentioned biomedical applications, the vast majority relate to the controlled release of drugs [61,75,77,78]. 

**Figure 10:**
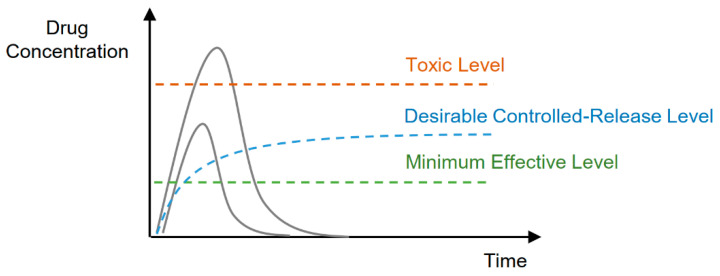
The graph presents the drug concentration over time connected with the levels of pharmaceutic effectivity.

The term ‘drug delivery’ means the methods or processes of administrating the pharmaceutical agent (drug) to achieve a therapeutic effect. Thus, the key factors, that shape the drug delivery system value is delivering the drug in the right area at the right time and in the right conditions. As a result, there are many factors that should be met to achieve efficient drug delivery. Additionally, the release rate of the drug must be also taken into account. It is a fact that achieving the desirable controlled release rate is quite hard. The concentration of the released drug can be either too low or too high, which results in, respectively, not achieving the effective therapeutics level or achieving the toxic level [79]. The schematic representation of the possible scenario is presented in Figure 10.

As a consequence, there is a need for creating ‘smart’ polymer biomaterials, responsive to the specified factor, that will be sensitive to various environmental stimuli (such as temperature, pH, or others). Some of the stimuli-responsive poly(2-oxazoline)-based biomaterials are presented in subsequent paragraphs, with descriptions of their biomedical applications.

Although polyoxazolines as homopolymers are characterized by pH- and thermo-responsive properties (most examples of using poly(2-oxazoline)s as stimuli-responsive polymers refer to these interactions), they are also included in copolymers that are sensitive to other types of external environmental impacts [75].

### 5.1. Temperature-Responsive Structures

Among the stimuli-responsive polymer systems, temperature is the most often employed factor. The temperature is an easily controllable and applicable stimulus for both in vitro and in vivo procedures [62]. The existence of a critical solution temperature is one of the distinctive characteristics of thermo-responsive polymers. The lower critical solution temperature (LCST) is the temperature below which a single phase exists in the (multi)polymer-solvent system. Above LCST, phase separation occurs by limiting the miscibility of the polymer in a particular solvent. Systems that become soluble upon heating have an upper critical solution temperature (UCST). These transition temperatures are the values obtained for the optimum concentration, since this parameter also influences the temperature at which the phase separation occurs. The LCST and UCST values are thus determined for a specific critical concentration of the polymer and are the property of the material. LCST and UCST are fundamental parameters of thermo-responsive polymers, which enable their application in biomedicine, e.g., for drug delivery. LCST and UCST values are often referred to as cloud point temperatures (T_CP_), which is not a material property (it is phase separation temperature, which is determined at a given moment for specific conditions). The vast majority of thermo-responsive polymers exhibit LCST behavior, which has been the subject of extensive research. Polymers with the LCST are miscible with the solvents only below a threshold temperature. It is worth mentioning that, for many polymers, the cloud point temperatures depend not only on the interactions of the polymer chains with the solvent molecules and their concentration but also on the presence of other components in the mixture. In the case of thermo-responsive polymers, the LCST value can be easily adjusted by the implementation of hydrophobic and hydrophilic (in an adequately selected ratio) moieties into the polymer structure [80,81,82,83,84,85].

A novel method to control the LCST of a polymer is to exploit the effect of molecular recognition. Modified polymers (introduction of a specific functional group) can form supramolecular bonds with particular molecules in solution. By varying the concentration of the solute, it is possible to tune the temperature at which phase separation occurs. In the following paper [86], the authors exploited this mechanism by modifying poly(2-oxazolines) with an adamantane group. The adamantane moiety formed inclusion complexes with β-cyclodextrin based on the guest–host interactions. By manipulating the concentration of β-cyclodextrin in the solution, the authors were able to achieve a very wide range of the LCST of the poly(2-oxazoline)-based copolymers (30–56 °C).

**Figure 11:**
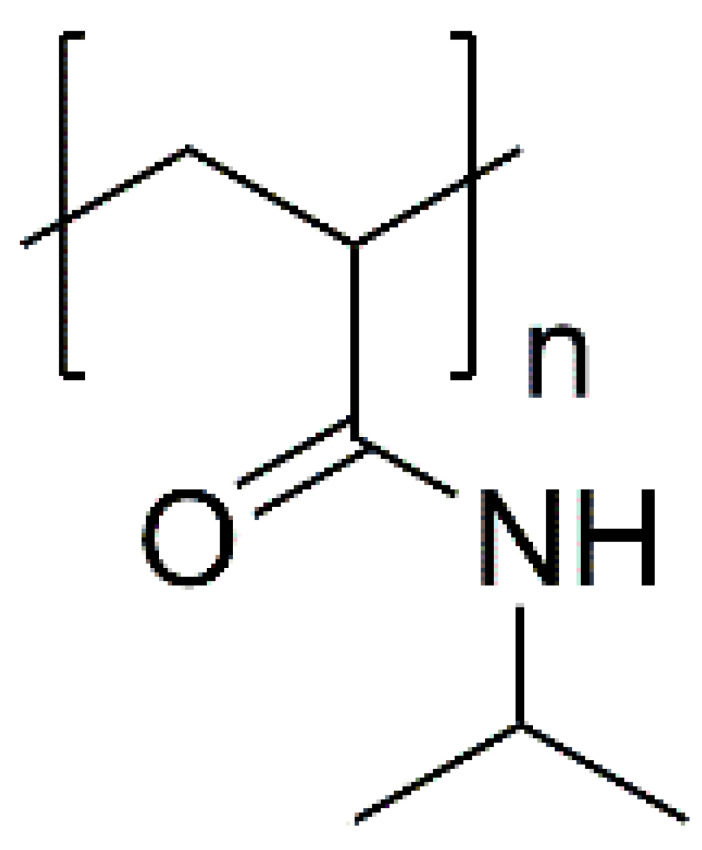
Poly(N-isopropylacrylamide) (PNIPAM).

**Figure 12:**
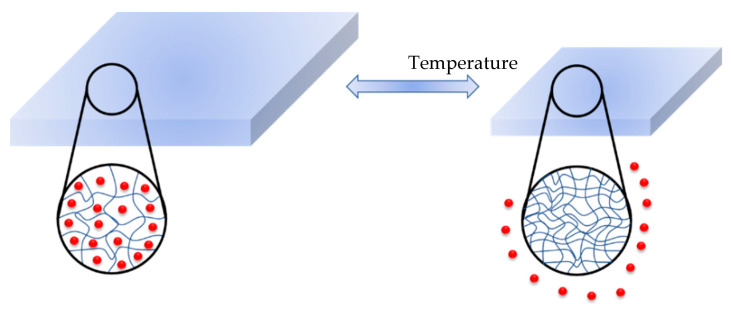
Shrink–swell behavior of a thermo-responsive hydrogel upon exposure to temperature. During the hydrogel shrinkage, the loaded drug is irreversibly released from the polymeric system. Adapted from [90].

In general, among the best-studied polymers in terms of thermal responsiveness are polymers based on poly(N-substituted acrylamide)s. The most well-studied representative of this group is poly(N-isopropylacrylamide) (PNIPAM) (Figure 11). Due to its lower critical solution temperature (LCST), which is less than 32 °C (near body temperature), PNIPAM-based hydrogels have been extensively employed to design thermo-responsive drug delivery systems. The hydrophilic moieties of PNIPAM form strong hydrogen bonds with water molecules at lower temperatures, which is responsible for its solubility in H_2_O. Above the LCST, the interactions associated with the hydrophobic isopropyl domain begin to predominate. As a consequence, the whole hydrogel structure shrinks, releasing water along with dissolved content (Figure 12). There is a lot of research confirming the use of this polymer for biomedical applications (controlled drug release, tissue engineering, etc.) [80,87,88,89].

**Figure 13:**
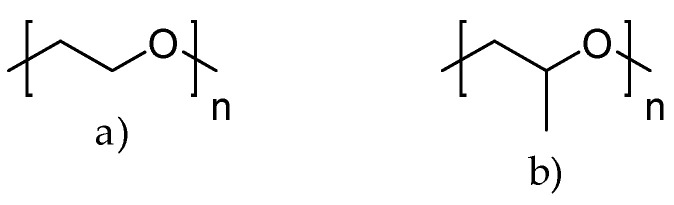
(**a**) Poly(ethylene glycol) (PEG) and (**b**) poly(propylene glycol) (PPG).

The self-organization of ordered structures in a solution is enabled by the combination of hydrophilic and hydrophobic monomers that are integrated into block copolymers, the most common of which are micelles. Micelles are helpful for encapsulating and delivering hydrophobic medicines into an aqueous environment. Well-studied thermo-responsive polymers that are capable of forming micelles are poly(ethylene glycol) (PEG) and poly(propylene glycol) (PPG) (Figure 13).

These polymers are often used as the hydrophilic part of amphiphilic copolymers. Stimuli-responsive micelles can be formed when amphiphilic moieties in the structure undergo micellization or demicellization by influencing the hydrophilicity/hydrophobicity balance, which can be adjusted by external factors, such as temperature. Another possibility is the formation of responsive micelles by incorporating some thermo-responsive monomers into the polymeric structure.

**Figure 14:**
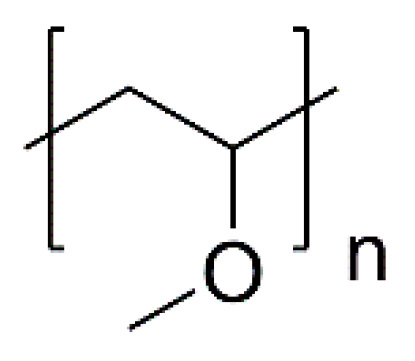
Poly(methyl vinyl ether) (PMVE).

Another example of thermo-responsive polymers is poly(vinyl ether)s (PVE). They display changes when temperature varies within the physiological temperature range, making this class of polymers potentially useful for biological applications. One of the most representative polymers from this group is poly(methyl vinyl ether) (PMVE) (Figure 14), which has a transition temperature of ca. 36 °C.

Thermosensitive copolymers of 2-ethoxyethyl vinyl ether and hydroxyethyl vinyl ether have a transition temperature of 20.5 °C and have been used, for instance, in the controlled release of vitamin E [91]. Other polymers that exhibit thermo-responsive behavior are poly(N-vinylcaprolactam) [92] and poly(phosphazene)s [93].

Generally, the thermo-responsive feature is a result of the previously mentioned lower critical solution temperature (LCST) behavior. It is known that the connection of the small hydrophobic side-chain to the polymeric backbone leads to thermo-responsive poly(2-oxazoline)s with LCST behavior. The range of polymers is limited by poly(2-methyl-2-oxazoline), a hydrophilic polymer, which does not show LCST behavior in water, and poly(2-butyl-2-oxazoline), with a hydrophobic butyl side chain, which causes insolubility in water. Additionally, the presence of hydrophobic aliphatic or aromatic side chains also results in obtaining hydrophobic poly(2-oxazoline) derivatives [94]. Some of the poly(2-oxazoline) homopolymers that exhibit LCST behavior and thus, show the thermo-responsive feature, are presented in Table 2.

Whereas the first thermo-responsive poly(2-oxazoline) analog is poly(2-ethyl-2-oxazoline) (PEtOx), reported by Lin and coworkers in 1988 [100], the polymeric analog with great potential seems to be poly(isopropyl-2-oxazoline) (PiPrOx). This polymer, first reported to be thermo-responsive by Uyama and Kobayashi in 1992 [97] seems to be the most studied and most popular thermo-responsive material [101,102], thanks to its LCST being similar to body temperature, 26 °C to 34 °C [103].

**Figure 15:**
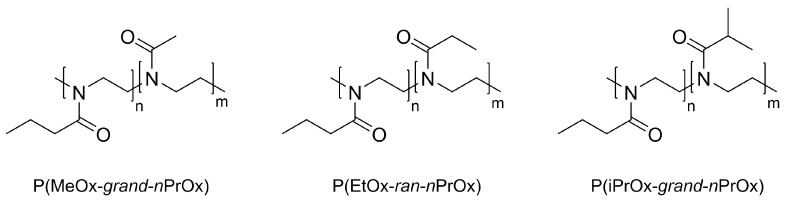
The structures of synthesized copolymers. Adapted from [14].

There are studies that focused on the examination of the properties of thermo-responsive-based materials, which can be successfully used for creating defined polymers with desired applications. One of them was reported in 2020 by Dworak and coworkers’ [104] investigation of the properties of gradient copolymers based on 2-isopropyl- and 2-*n*-propyl-2-oxazoline (poly(iPrOx-nPrOx)), as well as their temperature responsiveness and applicability as temperature-dependent-solubility (TDS) materials for cell culture in vitro. The investigation was based on the temperature-dependent cell detachment from the matrices due to temperature changes. This Polish research group has been investigating temperature-dependent materials [105]; thus, their study focusing on poly(2-oxazoline)-based thermo-responsive materials can be understood as a continuation and development of the previously reported work. The main parameter examined during this study was the behavior of the obtained matrices under aqueous conditions, which imitated the human body environment. From the obtained results, one of the greatest conclusions is that the presence of solid support for matrices with temperature-dependent solubility seems to be the crucial factor for their stability [104]. In a second study, reported in 2017 by Dworak and coworkers [14] a series of thermo-responsive copolymers were synthesized by a CROP reaction. The copolymers were based on 2-*n*-propyl-2-oxazoline and 2-ethyl-2-oxazoline or 2-*n*-propyl-2-oxazoline and 2-methyl-/2-isopropyl-2-oxazoline. The prepared copolymers were synthesized with the preservation of similar comonomer ratios. The obtained copolymers are presented in Figure 15.

**Figure 16:**
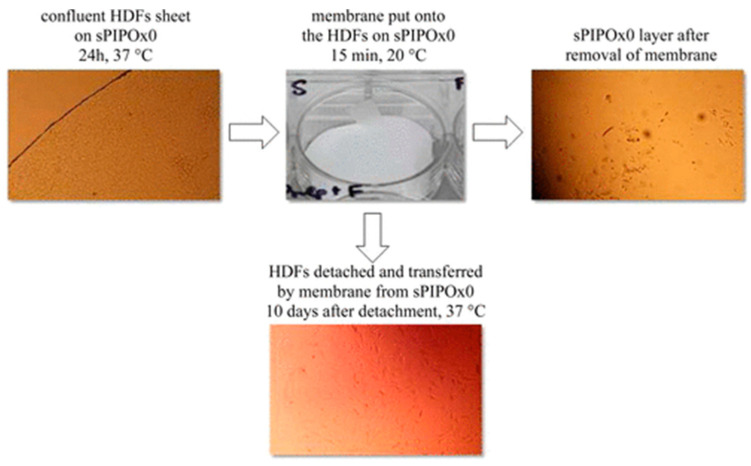
The representation of reported human dermal fibroblast (HDF) sheet detachment and transfer procedure. Reprinted from [107]. Copyright (2022), with permission from Elsevier [or applicable society copyright owner].

The reported studies were focused on the behavior of the obtained copolymers in aqueous solutions and thus, the influence of the concentration and composition of the copolymer on the final observed cloud points with subsequent aggregation, which was examined by the UV–Vis and dynamic-light-scattering measurements. As the results showed, the prepared copolymers displayed various aggregation behaviors shaped by not only the concentration but also by the temperature, as some parts of the examined copolymers were thought to be thermo-responsive. As Figure 16 shows, the obtained copolymers were different. The copolymerization of 2-*n*-propyl-2-oxazoline with 2-ethyl-2-oxazoline allows the obtention of random copolymers, whereas the CROP of 2-*n*-propyl-2-oxazoline with both 2-methyl-2-oxazoline or 2-isopropyl-2-oxazoline allows the obtention of gradient copolymers due to a greater difference in monomer reactivity. The obtained results showed that, for random copolymers, there is no hysteresis observed, and only the gradient copolymers at a specified concentration (5 mg mL^−1^) show the hysteresis of the phase transition. Additionally, the hysteresis was more evident for the copolymers obtained using 2-methyl-2-oxazoline. However, the self-arrangement of thermo-responsive 2-oxazoline gradient copolymers in water still requires more detailed studies and should be a subject of further research [14].

Another study that displays the direct exploitation of the thermo-responsive poly(2-oxazoline)-based materials for biomedical applications was notified in 2014 by Dworak and coworkers’ article on (co)poly(2-substituted-2-oxazoline) surfaces for dermal applications [106]. In this study, the thermo-responsive copolymers based on poly(2-substituted-2-oxazoline)s were synthesized to further prepare brush structures (linear polymer chains tethered on the surface) suitable for cell sheet engineering. The material was obtained by using biocompatible poly(2-isopropyl-2-oxazoline) and copolymers of 2-ethyl-2-oxazoline and 2-nonyl-2-oxazoline immobilized onto the modified surface of the glass using the grafting-to method. Additionally, in the first step, the copolymers were obtained by using CROP. The obtained surfaces were thermo-responsive, as their thickness and philicity varied due to the temperature changes. The temperature increase above the T_CP_ caused the shrinking of the polymer chains, whereas the decrease allowed noticing the layer thickness. The main application of prepared thermo-responsive surfaces is support for dermal fibroblast culture and detachment. Additionally, the adhesion and proliferation on the copolymer’s surfaces were observed only when the culture temperature achieved the T_CP_. The examined surfaces turned out to be suitable for the treatment of wounds and in skin tissue engineering thanks to their unique property of the detachment of the dermal fibroblast sheets from the polymer layers during the lowering of the temperature. This valuable feature could be also controlled by a variation in the temperature, without the necessity of mechanical or enzymatic methods for cell detachment [106].

Similar studies were also performed by Dworak and coworkers and reported in 2015 [107]. In this study, the application of different thermo-responsive poly(2-isopropyl-2-oxazoline) layers of different crystallite contents was investigated. As was reported, the main application of the proposed materials was intended to be cell sheet engineering. The obtained materials were applied as biomaterials, and the results showed the dependence between synthesized polymeric layer crystallinity and human dermal fibroblast adhesion, proliferation, and detachment (Figure 16).

As reported, after 24 h on culture plates, human dermal fibroblast formed sheets on all poly(2-isopropyl-2-oxazoline) layers. Additionally, the number of cells in the confluent sheet was doubled, which has been connected with the number of seeded cells. The influence of lowering the temperature was also examined. It was reported that, in the lower temperature environment, the morphology of the fibroblast changed, which was a result of the changes in the physicochemical properties of the layer. The morphology was changed from spindle-like to ellipsoidal. These morphology changes allow the detachment of the fibroblast sheet from the surface, resulting in the possibility of the detachment and transfer of cell sheets in the skin-substitute membrane [107]. The collected data allows the expansion of the knowledge of thermo-responsive materials for the biomedical engineering process and thus, can be treated as a prelude and milestone for the review article published in 2018 by the same Polish research group, mainly focusing on the application of thermo-responsive polymer surfaces for tissue engineering [108].

**Figure 17:**
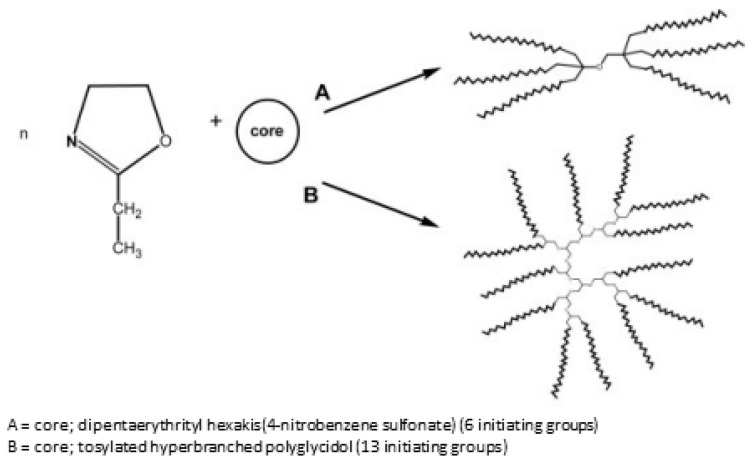
A schematic representation of the synthesis of star-shaped poly(2-ethyl-2-oxazoline)s. Reprinted from [38]. Copyright (2022), with permission from Elsevier [or applicable society copyright owner].

There was also star-shaped poly(2-ethyl-2-oxazoline)s synthesized, which differed in the length and number of arms from that reported in 2011 by Kowalczuk et al., a Polish research group [38]. As the scientific research group represented by Kowalczuk et al. was primarily focused on the synthesis of both thermo-responsive 2-oxazoline-based materials [104,109] and thermo-responsive star-shaped stable polymer nanolayers [110], the presented work may be treated as a sum of interests of the research group. Thus, the mentioned references can be regarded as a prelude to the final paper focusing on thermo-responsive star-shaped 2-oxazoline-based polymers, which were prepared by using dipentaerythrityl hexakis(2-nitrobenzene sulfonate) and a tosylated hyperbranched polymer of glycidol as initiators. The desired star shape was determined by nuclear magnetic resonance [38]. A schematic representation of the synthesis of the desired star-shaped materials is presented in Figure 17.

Because the obtained star-shaped polymers show a phase transition temperature in the range of 62 to 75 °C, the influence of molar mass and topological structure was established as a significant factor in temperature behavior, compared with the linear species. Additionally, due to the spherical shape combined with their thermo-responsive properties, the proposed materials might be useful for the controlled transport and release of various active compounds [38].

In studies reported by Polish scientists Kopka et al. in 2021, the application of poly(2-isopropenyl-2-oxazoline) was established [111]. As can be found in the literature, the poly(2-isopropenyl-2-oxazoline) (PiPOx) can successfully contribute to biomedicine due to its nontoxicity [112]. Another PiPOx advantage is its ability for a huge range of modifications with multiple chemical compounds containing desired functionalities. From the purpose of this review, the most important seems to be the post-polymerization modification reaction by using multiple aliphatic carboxylic acids, which directly leads to obtaining thermo-responsive copolymers [113,114]. The presented studies could thus be treated as a prelude for obtaining thermo-responsive hydrogel networks for biomedical applications. Generally, in the work reported by Kopka et al., a hydrogel network was prepared using poly(2-isopropenyl-2-oxazoline) (PiPOx), poly(ethylene oxide) (PEO), and selected biologically active compound: cinnamic acid, benzoic acid, or eugenol. The proposed networks, possessing in their structure compounds that demonstrate antimicrobial action, were thought to show antimicrobial activity, which may cause a huge interest in designing materials dedicated to biomedical applications. Due to the fact that skin damage, burns, and wounds are often associated with bacterial infections, there is a need for preparing dressings effective for common bacterial strains that caused skin infections [111]. Therefore, the proposed networks should be effective for *Staphylococcus aureus*, *Pseudomonas aeruginosa*, *Enterococcus spp.*, and *Escherichia coli*, which have been also responses to skin infection episodes [115,116]. During the synthesis, it was established that the one-step synthesis enabled efficient network formation with gel content at the 90% level. Additionally, the hydrogel formation and crosslinking stages were carried out without using any toxic compounds. The prepared hydrogels were investigated by measuring the swelling and mechanical properties. At least, after the characterization of prepared networks, the antimicrobial activity was also widely examined. It was reported that all of the synthesized hydrogels showed a desired antimicrobial effect in the contact zone. It should also be noted that the eugenol-loaded network showed a broader bacteriostatic effect inhibiting microorganism growth than the other hydrogels prepared during this work [111].

Furthermore, in 2015, Bouten et al. [99] reported an article focused on the synthesis and thermal properties of several thermo-responsive poly(2-oxazoline) derivatives containing a methyl ester side chain. The materials synthesized during this research were homopolymers based on 2-methoxycarbonylethyl-2-oxazoline (C2MestOx) and 2-methoxycarbonylpropyl-2-oxazoline (C3MestOx), as well as copolymers with 2-ethyl-2-oxazoline (EtOx) and 2-*n*-propyl-2-oxazoline (nPrOx), and varied by the composition. The mentioned methyl ester side chains were varied, and, as a result, there were 10, 20, 30, 50, and 70 mol % content established. The proposed research of T_CP_ of PC2MestOx and PC3Mest Ox was a pioneering investigation. As the obtained data showed, these T_CP_’s closely resemble the T_CP_’s of PEtOx and PnPrOx, respectively. Additionally, the obtained T_CP_ value of PC3MestOx was 26 °C; thus, the article showed that this polymer might be successfully used for many biomedical applications. Summarizing the results, almost all of the prepared materials showed that the T_CP_’s varied between 26 °C and 108 °C. The varied temperatures are related to the composition of the specified material [99]. This reported study can be helpful and might be treated as a prelude to further studies of the well-defined biomedical applications of the PC3MestOx-based materials.

### 5.2. pH-Responsive Structures

pH-Sensitive polymers contain moieties that may donate or absorb protons in response to a change in the pH of the surrounding environment. The electrostatic repulsion of the produced charges, brought on by the pH change, causes ionic interactions to influence the polymer chain in an aqueous solution to either lengthen or collapse. Polybases are capable of accepting protons under acidic conditions (expansion of the polymeric structure), whereas polyacids like poly(acrylic acid) donate their protons and swell under basic conditions. In other words, the term “pH-responsive polymers” refers to polyelectrolytes with weakly acidic or basic moieties that receive or donate protons when the pH level changes. In controlled release applications, pH is a common internal stimulant, since different pH values occur in different organs, tissues, and cellular compartments. In the case of poly(2-oxazolines), pH-responsiveness can be tuned with great efficiency by functionalization either by carboxylic acid or amines/amides [117]. As an example of incorporating the carboxylic group into the polyoxazoline chain, the post-polymerization functionalization of PC2MestOx can be proposed. In the following work [118] authors carried out the basic hydrolysis of the ester group in PC2MestOx with lithium hydroxide as the reagent. The reaction was followed by acidification with hydrochloric acid to obtain carboxylic groups along polymer chains. On the other hand, secondary amine groups can be attached into polyoxazoline chains by the partial acidic hydrolysis of any poly(2-oxazoline). Partially hydrolyzed poly(2-oxazoline)s can be further modified by reaction with methyl succinyl chloride, followed by amidation using primary amine. In such a way, it is possible to introduce amide groups into polymeric chains [119]. There are also reports indicating that poly(2-oxazoline)s with an ester group can be functionalized with hydrazine [120], as well as with a guanidinium or 2-aminoimidazole group [121]. pH-Responsive polymeric materials have been investigated in biomedical applications, primarily in controlled drug delivery in specific human organs or intracellular compartments (such as endosomes or lysosomes). Taking advantage of the fact that pathological changes in the human body often lead to changes in the physiological pH, pH-responsive polymers can be applied as controlled drug carriers. There are two main strategies for exploiting the properties of the pH-responsive materials: using polymers with ionizable groups that change their conformation and/or solubility in response to changes in the pH and designing polymers that contain functional groups sensitive to pH. After changing the pH of the environment, bonds that were formed between these functional groups and, for instance, drug molecules can be cleaved, resulting in releasing the previously bound medicine from the polymeric system. When ionizable groups are connected to the polymer structure, this results in a conformational shift for the soluble polymers and a change in the swelling behavior (in the case of hydrogels) [61,62,63,80].

As the previous subsection showed, the thermo-responsiveness of poly(2-oxazoline)-based systems are well-known and widely reported. However, pH-responsive materials with poly(2-oxazoline)s included are quite new in science and seem to be an area with great potential. The following studies report pH-responsive materials containing poly(2-oxazoline)s for biomedical applications.

**Figure 18:**
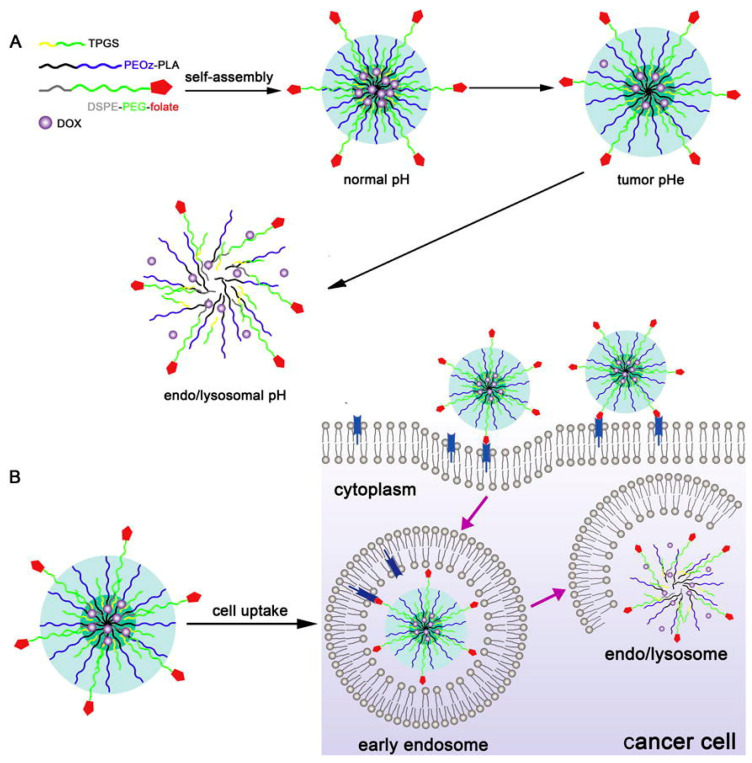
(**A**). The general overview of the synthesized copolymeric micelles modified with folate and the acid-triggered drug release from the pH-responsive micelles. (**B**). The general overview of synthesized copolymeric micelles for targeted and controlled drug delivery. Reprinted from [122]. Copyright (2022), with permission from Elsevier [or applicable society copyright owner].

One of the studies is reported in the 2015 article by Zhao et al. [122]. In this work, novel biocompatible poly(2-ethyl-2-oxazoline)-*co*-poly(_L, D_-lactide) (PEtOx-*co*-PLA) copolymers were synthesized. The self-assembly polymeric micelles were prepared by using the combinational technique, using both cationic and anionic ring-opening polymerization reactions. Subsequently, two chemotherapeutic agents were encapsulated (doxorubicin and D-alpha-tocopheryl polyethylene glycol 1000 succinate) to obtain a combinational material for treating multidrug resistance (MDR) cancer. These copolymers were used as potential pH-responsive biomaterials for tumor-targeted drug delivery systems and also controlled the delivery of anti-cancer drugs and the P-glycoprotein inhibitor. The efficient drug delivery by the synthesized micelles was the result of folate-mediated targeting, pH-responsive drug release, and D-alpha-tocopheryl polyethylene glycol 1000 succinate-aroused P-glycoprotein inhibition (Figure 18).

As reported, both of the proposed anticancer drugs were efficiently loaded into the self-assembled polymer micelles. It was estimated that the efficiency of MDR reversion for the micelles was confirmed by increasing doxorubicin accumulation combined with cytotoxicity. Additionally, there were some beneficial features noted. The observed benefits had a small size, high drug encapsulation efficiency, acidic pH-responsive quick-release characteristics, desirable tumor-targeting behavior, and cytotoxicity. Thus, the proposed multifunctional copolymeric micelles are suggested to be promising systems for improving MDR cancer treatment therapy [122].

Another study focused on using doxorubicin as an anti-cancer agent in poly(2-oxazoline)-based biomaterials was reported in a 2018 investigation by Hoelzer et al. [123]. In this paper, a co-polymer-based nanogel was synthesized by using block synthesis. The desired nanogel was prepared by cross-linking a copolymer micelle with a cationic 2-((4-)aminobutyl)-2-oxazoline (AmOx) core and 2-ethyl-2-oxazoline (EtOx) shell. As the first monomer applied during the synthesis was EtOx, the second one had to be introduced under an inert atmosphere to avoid the termination process prior to the extension of the polymer chain. The obtained poly-2-oxazoline-based nanogel micelles by using glutaraldehyde as a cross-linker were covalently loaded with doxorubicin using pH-responsive imine chemistry (Schiff base). From in vivo investigations, it has been found that treatment with the proposed nanogels revealed a significant tumor growth inhibition and also an increase in survival time. In comparison, pure doxorubicin alone showed no effect on tumor progression, which forms a strong suggestion that the proposed poly(2-oxazoline)-based nanogel micelles can be a promising strategy to control tumor growth with fewer side effects. Additionally, the biodistribution has also been investigated by the microscopy of mouse organs, and the obtained results showed the predominant localization of doxorubicin within tumorous tissue [123].

A different strategy for providing anti-cancer doxorubicin for breast tumor treatment was proposed by Gao et al. [124] in 2019. The proposed materials were pH-responsive dual drug co-delivery platforms for further combined chemo/photothermal therapies. The anti-cancer drug (doxorubicin) was loaded onto the coated by polydopamine black phosphorus nanosheets, to which poly(2-ethyl)-2-oxazoline was conjugated. Then, the loading into the surface of the nanocapsules of another anti-cancer agent (bortezomib) was conducted, which gave the proposed platforms the property of delivering two different drugs [124]. Bortezomib is a well-known antitumor agent applicable to patients with multiple myeloma; thus, it can successfully inhibit tumor cell growth by binding the threonine residues of active sites. However, it has to be mentioned that the application of bortezomib is limited, because of its non-specific binding to cell proteins, which causes it to be rapidly cleared by the blood that ultimately produces dose-related cytotoxicity [125]. The used polydopamine layer allows for enhancing the system’s stability before reaching the tumor site. It also maintains the photothermal effects for further modifications. On the other hand, the use of a pH-responsive system allows for remedying the deficiency of drugs in tumor therapy and increasing the anti-tumor effects thanks to high photothermal efficiency. The idea of the combination of several additional factors such as the surface charge from negative to positive at the tumor extracellular pH (~6.8), and irradiation under near-infrared laser causes the proposed platforms to be very promising for synergistic cancer therapy. Additionally, due to raising the drug loading content, cellular uptake, and pH-responsive release rate, these platforms exhibit high photothermal activity against tumor cells [124].

**Figure 19:**
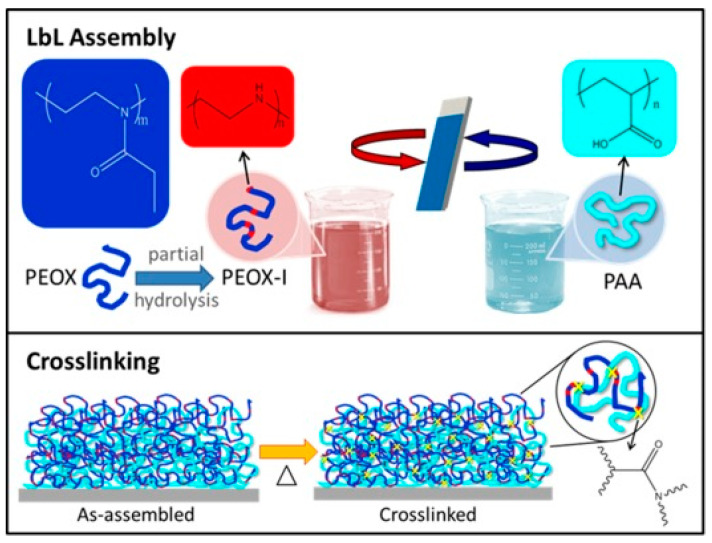
The general overview of the synthesis and thermal crosslinking process to obtain a pH-responsive coating from partially hydrolyzed poly(2-ethyl-2-oxazoline) (PEOx-I). Reprinted from [126]. Copyright (2022), with permission from Elsevier [or applicable society copyright owner].

The interesting strategy for the synthesis of pH-responsive anti-fouling layers has been reported in 2018 by Tan and coworkers, of whom the Polish scientist, Jańczewski, should be noticed [126]. In that report, poly(2-ethyl-2-oxazoline) (PEtOx) with polyacrylic acid (PAA) has been investigated to incorporate the PEtOx into controllable-thickness coatings. The multilayer thickness, studied during the presented report, varied from ~12 nm to ~1.5 µm. During synthesis, the partial hydrolysis of PEtOx has been introduced to achieve multilayer stabilization by heat-inducted crosslinking. As reported, the crosslinking process has been carried out at 150 °C for 40 minutes, which results in stabilization and counteracts the dissolution at pH > 4.5. Thus, above pH 4.5, the synthesized crosslinked coatings showed pH-responsive behavior. It has also been reported that the thermally crosslinked multilayers were stable against film loss and instead exhibited pH-responsive swelling. Additionally, the anti-fouling properties of the obtained multilayers have been investigated by evaluating the resistance to fouling by proteins, cells, and bacteria. The general overview of the chemical details of the presented report is presented in Figure 19.

In reported research, bovine serum albumin (BSA) was used as a model protein to study the resistance of prepared multilayers to protein adsorption properties. The obtained data showed that the crosslinked ~220 nm multilayers showed the highest effectivity toward BSA. However, the thinner or thicker multilayers showed increased BSA adsorption. Additionally, obtained layers of ~220 nm and above were effective in some of the bacterial strains, such as Gram-positive (*S. aureus*) and Gram-negative (*E. coli*) [126]. 

### 5.3. Light-Responsive Polymers

The majority of techniques for eliciting a response in stimuli-responsive polymeric systems rely on kinetically constrained diffusion. For hydrogels, for instance, that react to changes in pH or electrolyte concentration, there is a requirement for the transport of externally introduced ions in a polymeric system. The reaction of temperature-sensitive polymers can also be severely constrained by problems with heat transport. Because of this, many conventional stimuli-responsive polymers respond relatively slowly. The use of electric, magnetic, acoustic, or electromagnetic (light) fields provides a different kind of stimulation that gets around this problem [62,63,64,127,128].

When exposed to a particular wavelength and intensity of light, light-sensitive polymers can alter their physicochemical properties or break down. Light may be applied remotely, is frequently affordable, and can be used as a trigger to induce a specific reaction of the material. Usually, light responsiveness comes about as a result of structural changes in the polymer chains induced by light. The reason for this behavior is the presence of specific functional groups in the polymer backbone or side chains. Photo-irradiation is a very simple, non-invasive way to elicit responsive behavior, which is a key feature of photo-sensitive polymer systems. Practically the whole light spectrum, starting from the most energetic UV and extending all the way to the far infrared, may be used, providing a unique diversity compared to other types of stimuli. Moreover, electromagnetic wave irradiation is directed at a specific area or volume of the material. In light-responsive polymers, the effect on the relevant light-responsive moiety can be related to photoinduced isomerization and/or photochromism [67]

As examples of functional groups changing their characteristics under the influence of light, the following photo-responding groups can be mentioned: azobenzenes, spiropyrans, and spirooxazines. Among them, polymers with azobenzene groups have been investigated the most. Azobenzene is a chromophore that undergoes a rapid and complete change in electronic structure, geometric form, and polarity as a result of irradiation-induced *cis*-to-*trans* isomerization [64,128].

The majority of cases wherein numerous stimuli are impacted sequentially include the use of thermo-responsive polymers and light-responsive functional groups that are structurally connected to the polymer chain in order to tune the LCST [67].

**Figure 20:**
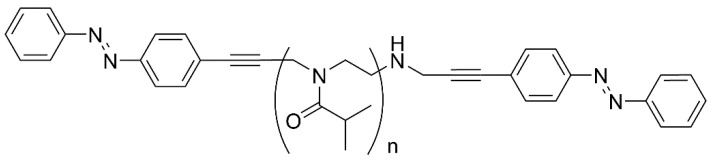
Thermo- and light-sensitive polymer based on poly(2-isopropyl-2-oxazoline).

The light-sensitive azobenzene group as an end-chain group was, for instance, used in a telechelic polyoxazoline-based (poly(2-isopropyl-2-oxazoline)) copolymer (Figure 20). Its synthesis and characterization was described in the following paper by Kim [129]. The LCST of the obtained polymer was impacted by this photo-induced trans–cis isomerization, with the polar cis form having a higher LCST (23 °C) than the nonpolar trans form (21 °C).

Another example of a similar approach that incorporates both light and thermal responsiveness is the following work [130]. The authors of the mentioned paper characterized a copolymer containing poly[oligo(2-ethyl-2-oxazoline)acrylate] and poly(2-nitrobenzyl acrylate) part (P(OEtOxA)-*co*-PNBA). By irradiation with UV light with a wavelength of 350 nm, they managed to increase the LCST of the copolymer by about 15 degrees. However, the design proposed by the authors assumes an irreversible structural change in the copolymer. It turned out to be, therefore, a one-time solution.

### 5.4. Magneto-Responsive Materials

The benefit of employing a magnetic field in the case of interactive materials depends on the variety of forms that the material responds to under a magnetic field, such as a magnetic guide under a permanent magnetic field, an increase in temperature by the action of a magnetic field, or both when employed alternately. Therefore, a variety of medication delivery pathways are possible with magnetically responsive systems. In general, magnetic guiding is achieved by injecting a magnetically sensitive carrier while concentrating an external magnetic field on the biological target. Due to enhanced drug accumulation within solid-tumor models, this idea has been shown to have significant potential in experimental cancer therapy. Magnetic core-shell nanoparticles are potential candidates for such a therapeutic strategy. According to the literature, the most popular magnetic particles for this purpose are ferric oxides, such as Fe_3_O_4_ and Fe_2_O_3_. After combining these inorganic compounds with polymers (mostly by coating), efficient magneto-responsive systems can be designed [63,64,131].

When magnetic nanoparticles are combined with hydrogels, as the result, new capabilities are created, such as magnetic-field-tunable mechanical properties. Notably, magnetic gels are often used in thermal treatment, wherein they are activated by switching magnetic fields and are intended to fight cancer cells. They can be utilized for controlled drug release when loaded with certain medicines. Additionally, magnetic hydrogels can imitate the contraction/elongation and electric capacity of soft human tissues [132].

One example of the successful synthesis of a material with magnetic properties based on poly(2-oxazoline) is the following work [133]. After the acetylation of β-CD, the EtOx monomer was grafted onto acetylated β-CD. Then the material was crosslinked with the amine-functionalized Fe_3_O_4_ nanoparticles. Fe_3_O_4_ nanoparticles, which have exceptional physicochemical and biological properties, are used as chemotherapeutic medication carriers. The implementation of magnetic-responsive nanoparticles to a polymer matrix is one of the ways to produce a magnetic-responsive platform for the drug delivery system. Doxorubicin hydrochloride (Dox) was loaded into the created magnetic nanohydrogel, and its drug loading and encapsulation effectiveness as well as its pH- and reduction-triggered drug release behaviors were examined.

A magnetic poly(2-oxazoline)-based hydrogel was synthesized by M. Cvek’s group [132] The poly(2-oxazoline)-based hydrogels were synthesized and characterized in this paper and can be used as a smart composite platform, useful in biomechanical or biomedical sectors, due to the unique combination of features manifested as excellent biocompatibility and sensitivity to magnetic fields.

### 5.5. Other Stimuli-Responsive Systems Based on 2-Oxazoline Derivatives

Due to the origination of both, exogenous and endogenous stimulus, they can be classified into the subsequent categories—chemical, physical, and biological. In comparison to the previous subsections, which are focused on the first two classes, this subsection will be dedicated to the third one [134]. As was mentioned, whereas the functioning of chemical stimulus is based on the molecular interaction changes between polymer chains [135], the physical stimulus influences the dynamics of polymer chains [136]. The functioning of a biological stimulus can be thus explained as the action of the biological agent (enzyme or receptor) triggering specific biochemical reactions or even ligand recognition, which results in the drug release [134]. These bioreducible drug delivery systems have already been widely reported, and they are successfully used within many systems such as anti-viral or even peptide delivery [137,138]. Generally, the reduction-responsive systems show high stability in systemic circulation, and, additionally, they are able to quickly respond to the differences in the concentrations of reducing species in specific environmental conditions. These beneficial features result in the improvement of the selectivity and efficiency of the entire treatment. It is also interesting that the major focus of stimuli-responsive systems has been to target cancer treatment, which is caused by the specific tumor microenvironment, such as low pH, elevated temperature, or redox level [134]. In general, the action of polymeric drug delivery systems sensitive toward the redox level (reduction-responsive polymeric systems) is based on taking advantage of the high gradient levels of glutathione (GHS), which are present in some types of cancer cells [139]. Additionally, whereas reductive stress is significantly higher inside cells than in the bloodstream, the reduction in induced drug release provides a controlled release mechanism after cellular uptake and thus, seems to be more interesting for biomedical applications [140].

**Figure 21:**
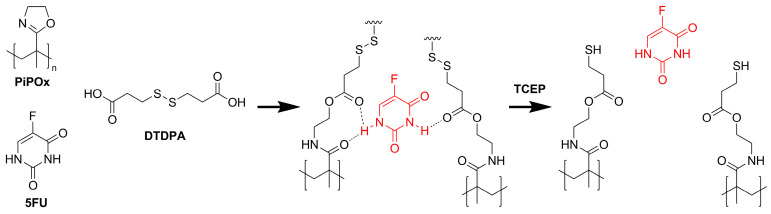
The general overview of the synthesis of poly(2-isopropenyl-2-oxazoline)-based molecularly imprinted polymers (MIPs) using 3,3’-dithiopropionic acid (DTDPA) as a reduction-responsive cross-linker and 5-fluorouracil (5FU) as a template behaviorist subsequent release in the presence of reductive reagent tris(2-carboxyethyl)phosphine) (TCEP). Reprinted from [140]. Copyright (2022), with permission from an open access Creative Commons CC BY 4.0 license.

The example of using the reduction-responsive poly(2-oxazoline)-based networks as materials for controlled drug release was reported in a 2020 study by Polish scientists Cegłowski et al. [140]. The report is focusing on the synthesis of poly(2-isopropenyl-2-oxazoline)-based molecularly imprinted polymers (MIPs) and their subsequent application in controlled drug release studies. Due to the fact that the used monomer, 2-isopropenyl-2-oxazoline, contains a double bond as the side group, and the polymerization of that 2-oxazoline derivative is quite different from the mechanism presented in the introduction part. The unusual behavior of the monomer during the polymerization leads to the polymer with a structure similar to poly(methacrylamide)s, with a 2-oxazoline ring at the side chain. To prepare MIPs, 3,3’-dithiopropionic acid (DTDPA) was used as a reduction-responsive cross-linking agent, whereas 5-fluorouracil (5FU) was used as a template, as this drug shows a broad activity spectrum against various tumors. The cross-lining process was performed in the presence of a template using the mentioned dicarboxylic acid possessing a disulfide bond. As the obtained MIPs’ structure contains disulfide bonds, the network is thought to show a reduction-triggered release in the presence of a specified reductive agent, represented by the tris(2-carboxyethyl)phosphine) (TCEP). The mechanism of the reduction-responsive release is based on the TCEP-inducting cleavage of the MIP cavities that directly leads to an increase in the release of the template. A general overview of the reported synthesis and released studies is presented in Figure 21 [140].

The obtained data showed that the synthesis of PiPOx-based MIPs is easy to perform and that the obtained materials show higher adsorption capacity than their non-imprinted analogs, which constitutes the success of the imprinting process. As the report was the first paper using the 2-isopropenyl-2-oxazoline monomer for MIP synthesis, the obtained data from the performed investigations are innovative and may be interesting for other scientific research groups. Except for the analytical studies (FT-IR, SEM, TGA, and DSC), the following examinations were performed to describe the adsorption mechanism, kinetics, and isotherms, as well as release studies in which the influence of TCEP addition was also tested. The results generally confirm the prediction that the PiPOx-based MIPs possessing disulfide bonds within the structure can be successfully used for the reduction-responsive 5FU release. The addition of TCEP caused the degradation of PiPOx–MIPs networks and thus, increase drug release as predicted. Additionally, the 5FU release from the examined materials (both MIPs and NIPs) was fitted with the Higuchi model at all examined pH values. The performed adsorption kinetic studies proved the difference in the mechanism of the adsorption of 5FU between PiPOx–MIPs and PiPOx–NIPs structures. To summarize, performed studies proved the efficiency of the proposed materials to be used in reduction-responsive localized anticancer therapy [140].

## 6. Conclusions

Stimuli-responsive polymers are a very important and promising group of materials that have many successful biomedical applications. A promising polymer group that stands out in this context are poly(2-oxazoline)s. These polymers are characterized by their high biocompatibility, hydrophilicity, and flexibility in the aspect of functionalization (incorporating the desired functional groups into their structure to change the properties).

This review presented the mechanisms causing the responsiveness of polymers to external factors. Due to developed synthesis technology and the wide variety of polymeric materials, it is possible to design systems based on an extensive assortment of physical and chemical stimuli. In addition to the most popular and well-known polymers based on temperature and pH control, there are also systems being investigated for the interaction of magnetic fields.

The use of poly(2-oxazolines) as the component of thermo- and pH-responsive polymeric systems in the context of controlled drug delivery as pristine polymers and polymer-based systems were described in detail. The examples cited in the review confirm that poly(2-oxazoline)s are excellent materials to be used in biomedical applications and that many scientists in Poland have had input into developments in this field of research. 

## Figures and Tables

**Table 1:** The chain transfer behavior of some of the functional groups of the CROP of 2-oxazoline monomers [12,21].

Strongly Interfering	Moderately Interfering	Non-Interfering
Mercaptans	Disulfides	Aromatic sulfides
Acids	Alkenes	Sulfones
Aromatic alcohols	Aliphatic alcohols	Aromatic nitrates
Aromatic aldehydes	Ketones	Esters
Thioesters	Acrylic secondary amides	Tertiary amides
Phenols	Primary amides	Secondary amides
Sulfoxides	Aromatic nitriles	Aromatic sulfides
	Aliphatic nitriles	Aliphatic chlorides

**Table 2:** The structures of thermo-responsive poly(2-oxazoline) homopolymers.

Polymer	LCST [°C] *	Characteristics	Refs.
poly(2-ethyl-2-oxazoline) PEtOx 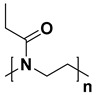	66 (for DP > 200)	It is an irreversible thermal transition when kept above its LCST for a long time. It is a very popular component in polyoxazoline-based copolymers	[95,96]
poly(isopropyl-2-oxazoline) PiPrOx 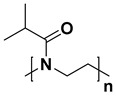	36	Due to its LCST being close to body temperature, it is one of the most prominent polymers regarding drug delivery applications. The LCST of this polymer, however, is strongly dependent on end groups.	[94,96,97]
poly(2-n-propyl-2-oxazoline) PnPrOx 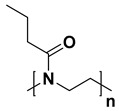	24 (for DP > 300)	Limited applications due to its low T_g_ and hydrophobicity	[95,96]
poly(2-cyclopropyl-2-oxazoline) PCPrOx 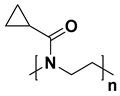	30 (for DP = 100)	High T_g_	[98]
poly(2-methoxycarbonylethyl-2-oxazoline) PC2MestOx 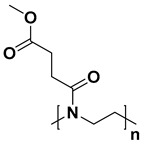	101 for DP = 100	Exceptionally high thermal transition temperature, susceptible to hydrolysis, low thermal stability compared to other poly(2-oxazolines), and relatively high T_g_	[99]
poly(2-methoxycarbonylpropyl-2-oxazoline) PC3MestOx 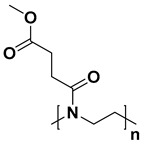	26	Susceptible to hydrolysis, andvery low T_g_	[99]

* In many cases, LCST depends highly on DP and the concentration of the polymer.

## Data Availability

The data presented in this study are available on request from the corresponding author.
